# *Serratia plymuthica* MBSA-MJ1 Increases Shoot Growth and Tissue Nutrient Concentration in Containerized Ornamentals Grown Under Low-Nutrient Conditions

**DOI:** 10.3389/fmicb.2021.788198

**Published:** 2021-12-02

**Authors:** Nathan P. Nordstedt, Michelle L. Jones

**Affiliations:** Department of Horticulture and Crop Science, Ohio Agricultural Research and Development Center, The Ohio State University, Wooster, OH, United States

**Keywords:** biofertilizer, biostimulant, floriculture, genome analysis, horticulture, nutrient stress, nutrient solubilization, PGPR

## Abstract

High fertilizer rates are often applied to horticulture crop production systems to produce high quality crops with minimal time in production. Much of the nutrients applied in fertilizers are not taken up by the plant and are leached out of the containers during regular irrigation. The application of plant growth promoting rhizobacteria (PGPR) can increase the availability and uptake of essential nutrients by plants, thereby reducing nutrient leaching and environmental contamination. Identification of PGPR can contribute to the formulation of biostimulant products for use in commercial greenhouse production. Here, we have identified *Serratia plymuthica* MBSA-MJ1 as a PGPR that can promote the growth of containerized horticulture crops grown with low fertilizer inputs. MBSA-MJ1 was applied weekly as a media drench to *Petunia*×*hybrida* (petunia), *Impatiens walleriana* (impatiens), and *Viola*×*wittrockiana* (pansy). Plant growth, quality, and tissue nutrient concentration were evaluated 8weeks after transplant. Application of MBSA-MJ1 increased the shoot biomass of all three species and increased the flower number of impatiens. Bacteria application also increased the concentration of certain essential nutrients in the shoots of different plant species. *In vitro* and genomic characterization identified multiple putative mechanisms that are likely contributing to the strain’s ability to increase the availability and uptake of these nutrients by plants. This work provides insight into the interconnectedness of beneficial PGPR mechanisms and how these bacteria can be utilized as potential biostimulants for sustainable crop production with reduced chemical fertilizer inputs.

## Introduction

The plant rhizosphere, the microscopic environment surrounding plant roots, hosts a diversity of microorganisms. Plants secrete a variety of root exudates into the rhizosphere including sugars, vitamins, and amino acids, which assist in recruiting specific microbial populations (Lugtenberg and Kamilova, 2009; Martín-Sánchez et al., 2020). In return, many of these microbes play essential roles in the rhizosphere ecosystem by influencing both plant and soil health (Vessey, 2003). The study of these beneficial plant growth promoting rhizobacteria (PGPR) and their contribution to agriculture has gained significant interest in recent years (Bhardwaj et al., 2014). PGPR can stimulate plant growth through a variety of mechanisms including modulating phytohormone levels, inhibiting growth of plant pathogens, producing secondary metabolites, and increasing the availability and uptake of nutrients for their plant host (Raklami et al., 2019; Martín-Sánchez et al., 2020).

Plants require 14 essential mineral nutrients to support proper growth and development, and agricultural systems often rely on the addition of chemical fertilizers to provide adequate nutrients to support crop production (Marschner, 2011). However, high rates of fertilizer are frequently attributed to surface and groundwater pollution due to nutrient leaching and runoff (Adesemoye and Kloepper, 2009). The bioavailability of nutrients for plant uptake plays a significant role in plant nutrient use efficiency (NUE), and therefore, the lack of bioavailability increases the susceptibility of nutrients to leaching and runoff (Adesemoye and Kloepper, 2009; Bindraban et al., 2015). This is of particular concern for horticulture crops grown in peat-based soilless substrates, which have lower ion exchange capacities than field soil, increasing the possibility that nutrients will be removed from the substrate *via* leaching (Bachman and Metzger, 2008). Therefore, it is important that crop producers find sustainable ways to produce crops with high yield and quality while minimizing chemical fertilizer inputs.

Studies have shown that the application of PGPR can increase the NUE of plants, thereby reducing the amount of nutrients that are leached into the environment (Khan, 2005; Adesemoye et al., 2008, 2010; Shaharoona et al., 2008; Arif et al., 2017; Pereira et al., 2020). A meta-analysis of studies evaluating PGPR application on plant NUE showed an average increase in NUE of 5.8kg yield per kg nitrogen fertilizer applied (Schütz et al., 2018). Direct mechanisms that PGPR can utilize to facilitate increased nutrient bioavailability and uptake by plants include nitrogen fixation, nutrient solubilization, sulfur oxidation, and chelation of metals (Lugtenberg and Kamilova, 2009).

The availability of essential plant nutrients including phosphorus, potassium, and zinc is dependent on their solubility in the rhizosphere (Ruzzi and Aroca, 2015). When these nutrients are bound by other salts and metals, plants are unable to access them for growth and development. However, bacterial solubilization can make them readily accessible for plant utilization and less likely to be removed from the growing substrate *via* leaching (Adesemoye and Kloepper, 2009). Many bacterial species that are plant growth promoters have been identified as efficient solubilizers of phosphorus, potassium, and zinc (Sharma et al., 2013, 2016; Kamran et al., 2017; Borgi et al., 2020). The application of phosphorus-solubilizing *Bacillus* and *Aspergillus* strains reduces the level of fertilizer required for optimal yield and tissue nutrient content in strawberry (Güneş et al., 2014). Although many different bacterial enzymes can solubilize these nutrients, bacterial-produced organic acids are thought to be the primary mechanism involved in increasing the availability of insoluble phosphorus, potassium, and zinc (Rodríguez et al., 2006; Hussain et al., 2015; Parmar and Sindhu, 2019).

Iron is an essential nutrient for both plants and bacteria; however, it is not readily available in the required concentrations in its predominant form as a ferric ion (Gupta et al., 2015). Bacteria can produce siderophores that act as chelating agents to make the element available for uptake by plants (Crowley et al., 1988; Sharma et al., 2003; Lurthy et al., 2020). Additionally, siderophores can act similarly to chelate other essential metals, such as copper (Yoon et al., 2010). Application of the siderophore-producing PGPR *Chryseobacterium* C138 increased the iron content of tomato plants supplied solely with ferric iron (Radzki et al., 2013). Siderophore production by PGPR allows for reduced chemical inputs to meet the demand of available iron to plants (Gupta et al., 2015).

It is advantageous to crop producers and the environment that PGPR increase the availability or uptake of multiple essential plant nutrients, allowing for a reduction in total fertilizer inputs. Formulation of these PGPR into biostimulant products would then make them widely available for commercial greenhouse operations. Therefore, in this work, we have evaluated the model PGPR strain *Serratia plymuthica* MBSA-MJ1 for its ability to increase plant growth and tissue nutrient concentration of three economically important horticulture crop species produced with low fertilizer inputs. MBSA-MJ1 was previously evaluated for its ability to increase plant growth during recovery from severe water stress (Nordstedt and Jones, 2021). In addition, we have characterized the *in vitro* and genomic characteristics of this PGPR to begin elucidating different putative mechanisms used to promote plant growth with an emphasis on nutrient availability. This work demonstrates the potential of this PGPR as a commercial biostimulant, allowing growers to reduce chemical fertilizer inputs without sacrificing crop health or quality.

## Materials and Methods

### Bacterial Strain

*Serratia plymuthica* MBSA-MJ1 originated from a bacteria collection in the laboratory of Dr. Christopher Taylor (Aly et al., 2007), although the environmental source of this strain is unknown. MBSA-MJ1 was recently evaluated for its ability to reduce *Botrytis cinerea* infection in *Petunia*×*hybrida* (South et al., 2020) and to stimulate the growth of plants recovering from severe water stress (Nordstedt and Jones, 2021).

### Low-Nutrient Greenhouse Trial

A previously established greenhouse trialing protocol was utilized to evaluate the ability of *S. plymuthica* MBSA-MJ1 to increase the growth of three economically important ornamental plant species produced with low fertilizer inputs (Nordstedt et al., 2020). *Petunia*×*hybrida* ‘Picobella Blue’ (petunia; Syngenta Flowers Gilroy, CA), *Impatiens walleriana* ‘Super Elfin Ruby’ (impatiens; PanAmerican Seed, West Chicago, IL), and *Viola*×*wittrockiana* ‘Delta Pure Red’ (pansy; Syngenta Flowers) seeds were sown in Pro-Mix PGX soilless media (Premier Tech Horticulture, Quakertown, PA) and grown for 3weeks. Seedlings were then transplanted to 11.4cm diameter pots containing Pro-Mix PGX. All plants were fertilized with 25mgL^−1^ N from 15N–2.2P–12.5K–2.9Ca–1.2Mg water soluble fertilizer (JR Peters Inc., Allentown, PA) at each irrigation to provide low-nutrient conditions. Plants were arranged in a Randomized Complete Block Design by species. Each block contained one bacterial-treated and one untreated control plant with *n*=13 for petunia, *n*=14 for pansy, and *n*=18 for impatiens. Greenhouse temperatures were set at 24/18°C (day/night) and supplemental lighting was provided by high-pressure sodium and metal halide lights (GLX/GLS e-systems GROW lights, PARSource, Petaluma, CA, United States) to maintain light levels above 250mmolm^−2^ s^−1^ and provide a 16h photoperiod.

Each plant was treated weekly with 120ml working inoculum of *S. plymuthica* MBSA-MJ1. Working inoculum was prepared by diluting an overnight culture grown in LB media (OD_595_=0.8) 1:100 in reverse osmosis (RO) water as described previously (Nordstedt et al., 2020). Diluted uninoculated LB media was used as the control. Plants were grown under low-nutrient conditions with weekly bacterial treatments for 8weeks, at which point all plants had open flowers. Plant performance was then evaluated by counting flower number (open flowers and flower buds showing color) and collecting shoots (including stems and leaves) and flowers for biomass measurements. Shoot and flower biomass was combined to get the total shoot biomass. Potting media was removed from the roots by rinsing in water, and root tissue was collected separately from shoot tissue. Roots, shoots, and flowers were dried at 49°C for at least 96h and then weighed to determine dry weights. Root dry weights and shoot dry weights were used to calculate the root:shoot ratio.

### Tissue Nutrient Concentration

Dried leaf and stem tissue (shoots minus the flowers) collected from the low-nutrient greenhouse trial were pooled for tissue nutrient analysis. Each sample consisted of tissue pooled separately for each plant species and treatment (MBSA-MJ1 and control) and contained tissue from three blocks located spatially together in the greenhouse (*n*=3). Tissue nutrient analyses were conducted at the Service Testing and Research Laboratory (The Ohio State University/OARDC, Wooster, OH). Total tissue nitrogen (N) concentration was determined from a 100mg sample using the Dumas combustion method (Vario Max combustion analyzer, Elementar America, Inc., Germany; Sweeney, 1989). The phosphorus (P), potassium (K), magnesium (Mg), calcium (Ca), sulfur (S), boron (B), copper (Cu), iron (Fe), sodium (Na), molybdenum (Mo), manganese (Mn), and zinc (Zn) concentration of the tissue was then determined by inductively coupled plasma spectrometry (model PS3000, Leeman Labs Inc., Hudson, NH) of a 250mg tissue sample following nitric acid microwave digestion (Discover SP-D, CEM Corporation; Isaac and Johnson, 1985).

### *In vitro* Characterization

#### Sole Carbon Source Utilization

Growth of *S. plymuthica* MBSA-MJ1was evaluated on media containing different sole sources of carbon, including cellobiose, fructose, galactose, glucose, glycerol, mannitol, sucrose, and ribose. Basal media was prepared according to Pridham and Gottlieb (1948) supplemented with 1% w/v (1% w/w for glycerol) of each carbon source. Cells from an overnight culture of MBSA-MJ1 were resuspended in PBS buffer, and 5μl of inoculum was struck out into each carbon source plate in triplicate (*n*=3). Plates were incubated at 28°C for 96h and growth on each carbon source was recorded as yes/no.

#### Phosphate Solubilization

Phosphate solubilization capabilities of *S. plymuthica* MBSA-MJ1 were evaluated according to Mehta and Nautiyal (2001). An overnight culture grown in LB media was diluted to OD_595_=0.2 and 80μl was used to inoculate 8ml NBRIP media in triplicate (*n*=3). Uninoculated LB in NBRIP media was used for the negative control. NBRIP media consisted of (per liter): 10.0g glucose, 5.0g Ca_3_(PO_4_)_2_, 5.0g MgCl_2_ • 6H_2_O, 0.25g MgSO_4_ • 7H_2_O, 0.2g KCl, and 0.1g (NH_4_)_2_SO_4_. The pH of the media was adjusted to 7.0 before autoclaving. Following inoculation, samples were incubated at 30°C for 72h with 200rpm shaking. After incubation, samples were centrifuged at 4°C and 5,000rpm for 10min to collect bacterial cells and remaining insoluble phosphate. The supernatant was collected, and the pH was measured as an indicator of solubilized phosphate.

#### Potassium Solubilization

The potassium solubilization ability of *S. plymuthica* MBSA-MJ1 was evaluated according to a modified protocol adapted from Rajawat et al. (2016). Cultures were prepared similar to those described above for the phosphate solubilization assay, and 80μl was used to inoculate 8ml Aleksandrov (AKV) liquid media. Uninoculated LB in AKV media served as the negative control. AKV media consisted of (per liter): 5.0g glucose, 2.0g Ca_3_(PO_4_)_2_, 0.5g MgSO_4_ • 7H_2_O, 0.1g CaCO_3_, 0.005g FeCl_3_, and 2.0g montmorillonite as the insoluble form of potassium. The pH of the media was adjusted to 7.2 before autoclaving. After inoculation, samples were incubated at 30°C for 72h with 200rpm shaking. Following incubation, bacterial cells and insoluble montmorillonite were collected by centrifugation at 4°C and 5,000rpm for 10min, and the supernatant was collected. The pH of the supernatant was measured as an indicator of solubilized potassium.

#### Quantification of Solubilized Phosphate and Potassium

Total solubilized orthophosphate and potassium in defined media were measured as validation of the nutrient solubilization capabilities of *S. plymuthica* MBSA-MJ1. Cultures were grown and inoculated in NBRIP and AKV media to evaluate phosphate and potassium solubilization, respectively. Samples were prepared similar to the solubilization assays, and after incubation and centrifugation, the supernatant was filtered through a 0.45μm nylon filter and total orthophosphate (PO_4_) or potassium in the filtrate was determined using Dionex ICS-6000 Ion Chromatograph (Thermo Fisher Scientific Inc., Waltham, MA).

### Iron and Copper Chelation

The iron and copper chelation capabilities of *S. plymuthica* MBSA-MJ1 were evaluated according to Schwyn and Neilands (1987). All labware for media preparation and culturing the samples was washed in a 6M HCl acid bath for at least 24h prior to use. Succinic acid (SA) media consisted of (per liter): 6.0g K_2_HPO_4_, 3.0g KH_2_PO_4_, 1.0g (NH_4_)_2_SO_4_, 0.2g MgSO_4_ • 7H_2_O, and 4.0g succinic acid disodium salt, and the pH of the media was adjusted to 7.0 before autoclaving. The chrome azurol S (CAS) assay solution was prepared in two solutions. Solution one contained 20ml H_2_O, 6.0ml 10mm hexadecyltrimethylammonium bromide, 7.5ml 2mm CAS, and 1.5ml 1mm FeCl_3_ or CuCl_2_ to test for iron and copper chelation, respectively. Solution two contained 4.307g anhydrous piperazine in 30ml H_2_O, pH adjusted to 5.6 with 12M HCl. Once prepared, solution one was added to solution two and the final volume was brought to 100ml with H_2_O to make up the working CAS assay solution. The CAS assay solution was stored in a dark polyethylene bottle.

For the assay, 5ml SA media was inoculated with a single colony of MBSA-MJ1 and incubated for 24h. After incubation, cultures were diluted to OD_595_=0.5, and 80μl diluted culture was inoculated in 8ml SA media in triplicate (*n*=3). Uninoculated SA media was used as the negative control. Samples were incubated at 28°C for 24h with 200rpm shaking. After incubation, bacterial cells were collected by centrifugation at 4°C and 5,000rpm for 10min. After centrifugation, 750μl supernatant was mixed with 750μl CAS assay solution and incubated in the dark for 15min with 100rpm shaking to allow for color development. Absorbance of each sample was then measured at 630nm, with a decrease in absorbance corresponding to chelation of the respective metal and a color change from blue to orange. The chelation percentage of the MBSA-MJ1 sample was calculated using [(*Ac*-*Ab*)/*Ac*]×100%, where *Ac* is the absorbance at 630nm of the control sample, and *Ab* the absorbance at 630nm of the MBSA-MJ1 sample (Dimkpa, 2016).

### Statistical Analysis

Statistical analyses for the greenhouse trial and *in vitro* characterization experiments were conducted in R Studio version 3.5.2 using an ANOVA with the model: *Y*=μ+treatment+block. The Tukey’s HSD test was used to determine statistical significance between MBSA-MJ1 treatment and the negative control. For the greenhouse trial, each plant species was analyzed independently of each other.

### Genome Analyses

Genome sequence data for *S. plymuthica* MBSA-MJ1 (Nordstedt and Jones, 2021) were used to search for genes putatively involved in increasing nutrient availability, carbon and amino acid metabolism, and heavy metal resistance. Genome sequence data can be found in the National Center for Biotechnology Information data base accession # PRJNA669647. AntiSMASH (v 5.0) was used to identify secondary metabolite biosynthetic gene clusters within the genome of MBSA-MJ1 (Blin et al., 2019). BlastKOALA was used for annotation of KEGG pathways involved in nutrient transport, amino acid synthesis, and carbon metabolism and transport (Kanehisa et al., 2016).

## Results

### Low-Nutrient Greenhouse Trial

**Figure 1:**
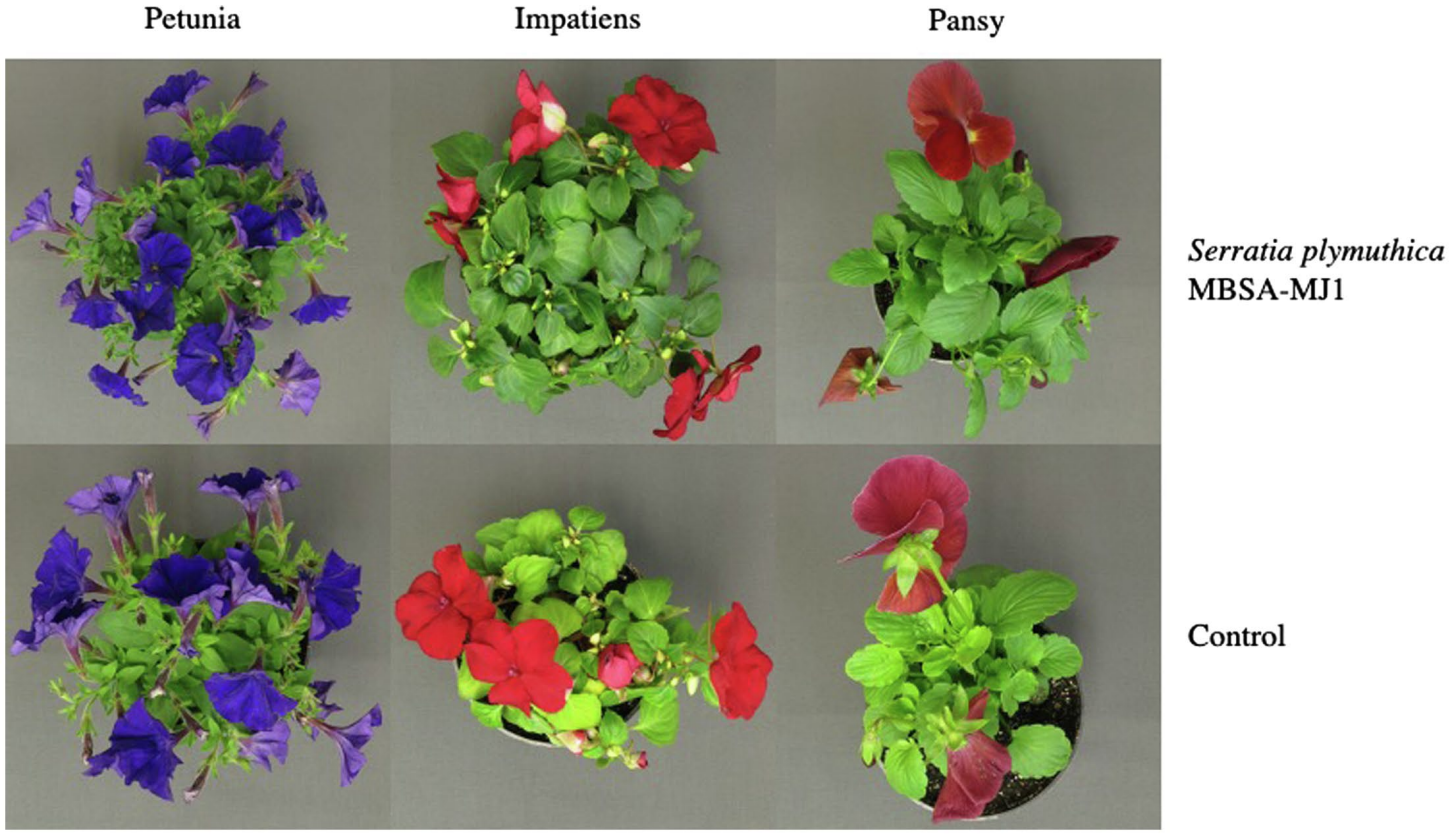
*Petunia*×*hybrida* (petunia), *Impatiens walleriana* (impatiens), and *Viola*×*wittrockiana* (pansy) plants grown under low-nutrient conditions had increased visual quality when treated with *Serratia plymuthica* MBSA-MJ1. Plants were treated weekly for 8weeks with *S. plymuthica* MBSA-MJ1 or uninoculated LB (control) as a media drench and fertilized at each irrigation with 25mgL^−1^ N from 15N–2.2P–12.5K–2.9Ca–1.2Mg water soluble fertilizer to induce low-nutrient conditions.

**Figure 2:**
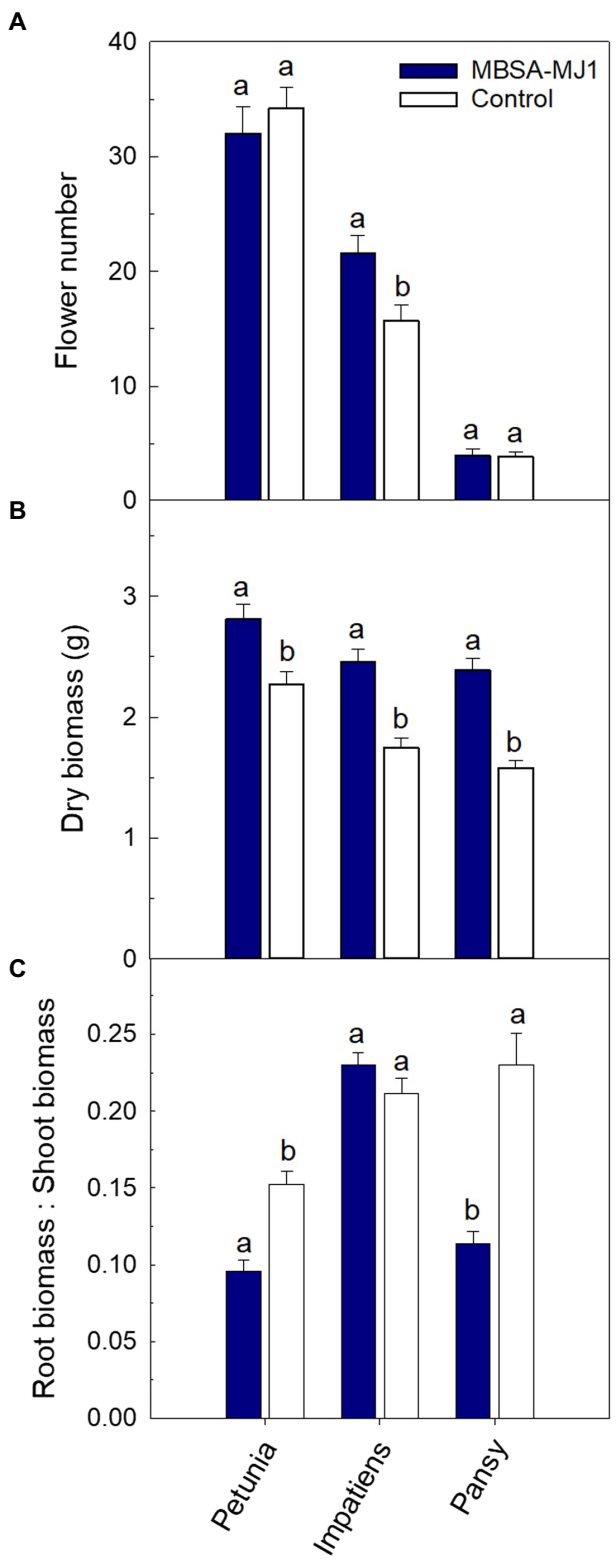
Treatment with *S. plymuthica* MBSA-MJ1 influenced plant growth parameters for *Petunia*×*hybrida* (petunia), *Impatiens walleriana* (impatiens), and *Viola*×*wittrockiana* (pansy) plants grown under low-nutrient conditions. Plants were treated weekly with a media drench of *S. plymuthica* MBSA-MJ1 (blue bars) or uninoculated LB (control; white bars). Total number of flowers **(A)** and total shoot biomass (dry weight) **(B)** was measured 8weeks post-transplant. The root:shoot ratio **(C)** was calculated with root and total shoot dry weights. Bars represent mean (±SE) with different letters representing significant differences (*p*<0.05), and *n*=13 for petunia, *n*=14 for pansy, and *n*=18 for impatiens.

Application of *S. plymuthica* MBSA-MJ1 increased the visual quality of all three plant species. Overall, plants treated with MBSA-MJ1 were larger and had greener leaves than the control plants that were not treated with bacteria (Figure 1). In addition to increasing the visual quality of plants, impatiens treated with MBSA-MJ1 had significantly more flowers than the uninoculated control, an average increase of six flowers per plant (Figure 2A). Application of MBSA-MJ1 significantly increased the total shoot biomass of all three plant species. Petunia, impatiens, and pansy grown under low-nutrient conditions and treated with MBSA-MJ1 had 24, 41, and 51% greater biomass, respectively (Figure 2B). Petunia and pansy plants treated with MBSA-MJ1 also had significantly lower root:shoot biomass ratio, whereas there was no statistically significant difference in impatiens (Figure 2C).

### Tissue Nutrient Concentration

**Figure 3:**
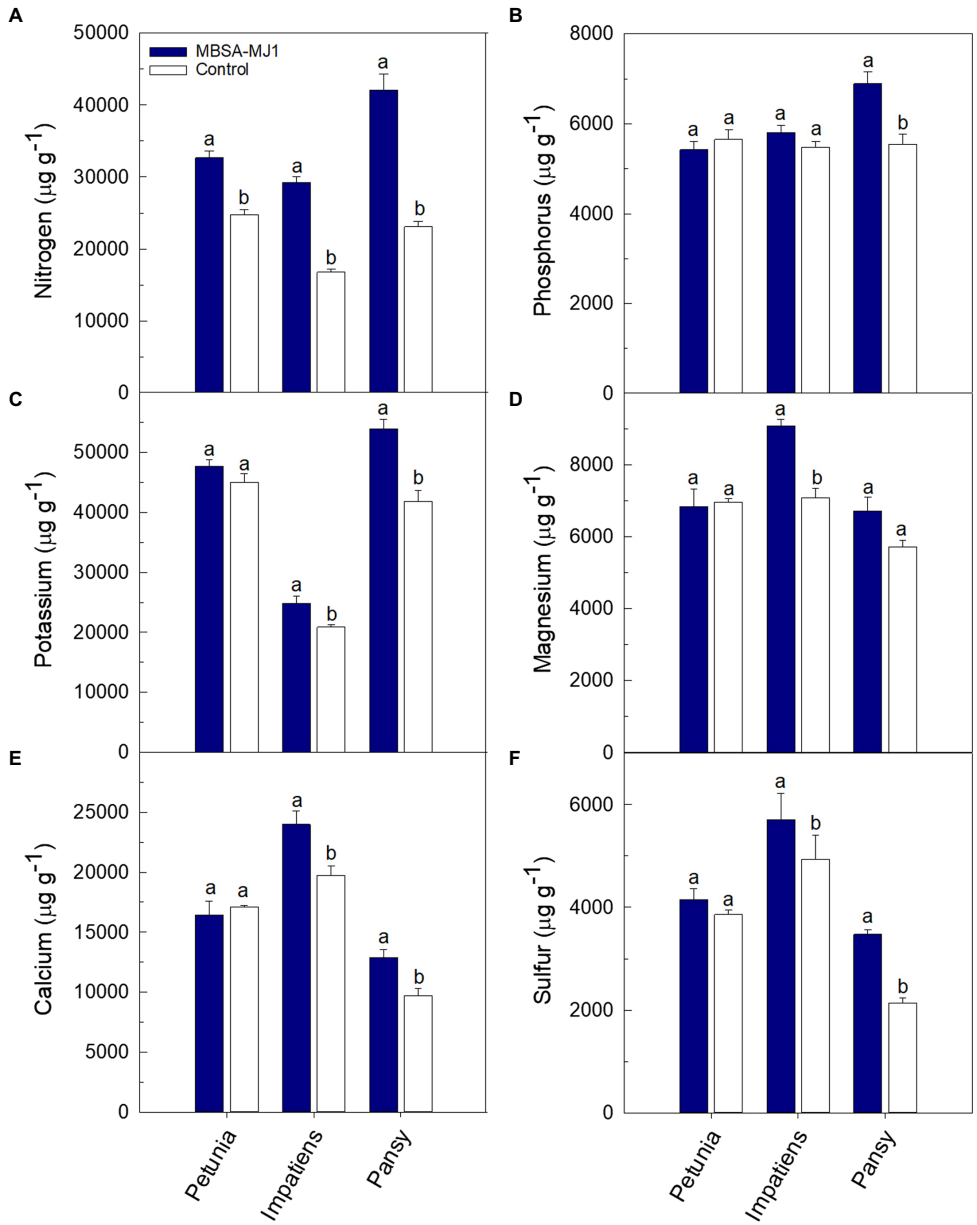
Tissue macronutrient concentration of *Petunia*×*hybrida* (petunia), *Impatiens walleriana* (impatiens), and *Viola*×*wittrockiana* (pansy) plants grown under low-nutrient conditions: nitrogen **(A)**, phosphorus **(B)**, potassium **(C)**, magnesium **(D)**, calcium **(E)**, and sulfur **(F)**. Plants were treated weekly with a media drench of *S. plymuthica* MBSA-MJ1 (blue bars) or uninoculated LB (control; white bars). Tissue nutrient concentration was evaluated 8weeks post-transplant. Bars represent the mean (±SE) with different letters representing significant differences (*p*<0.05) and *n*=3.

**Figure 4:**
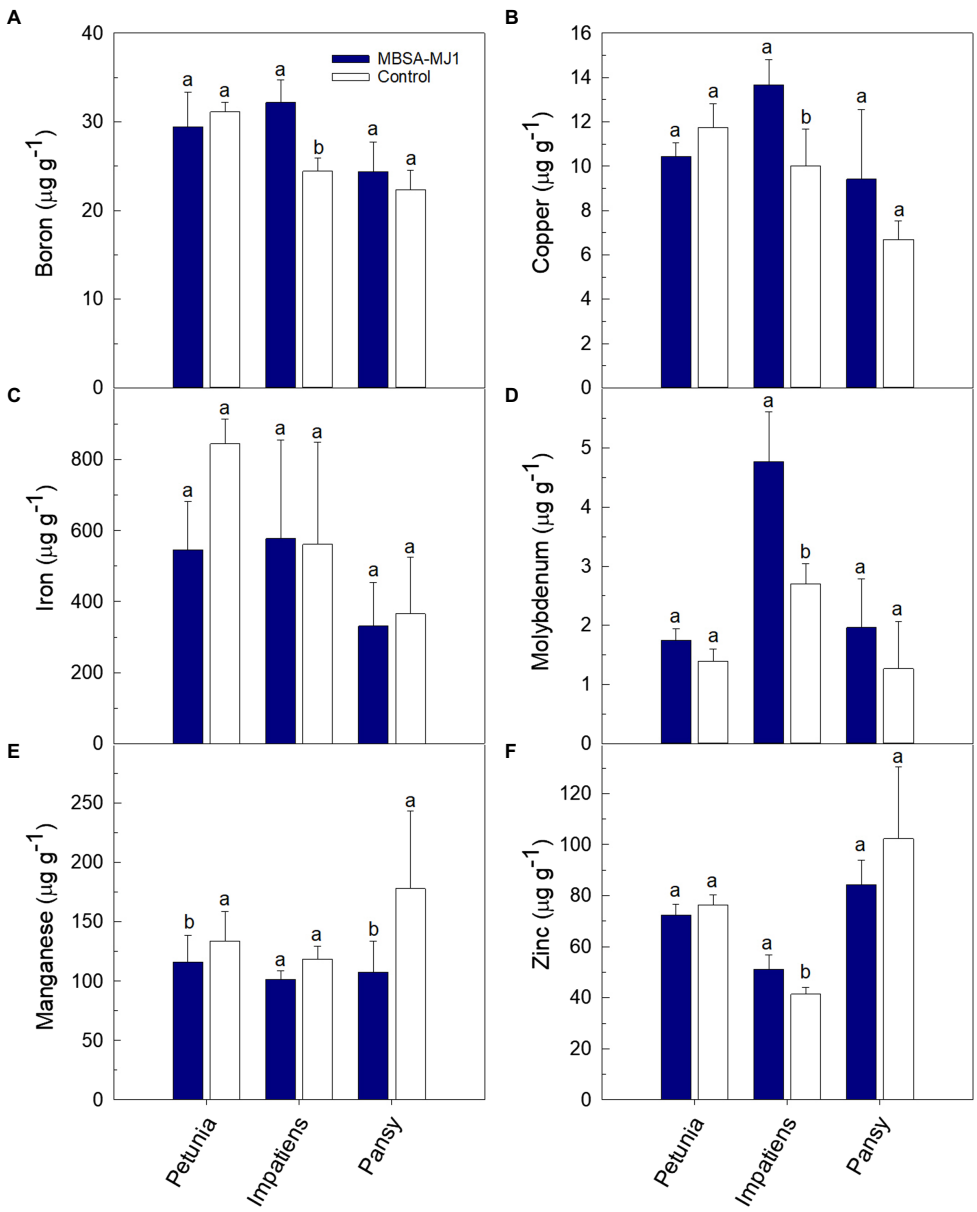
Tissue micronutrient concentration of *Petunia*×*hybrida* (petunia), *Impatiens walleriana* (impatiens), and *Viola*×*wittrockiana* (pansy) plants grown under low-nutrient conditions: boron **(A)**, copper **(B)**, iron **(C)**, molybdenum **(D)**, manganese **(E)**, and zinc **(F)**. Plants were treated weekly with a media drench of *S. plymuthica* MBSA-MJ1 (blue bars) or uninoculated LB (control; white bars). Tissue nutrient concentration was evaluated 8weeks post-transplant. Bars represent the mean (±SE) with different letters representing significant differences (*p*<0.05) and *n*=3.

Plants grown under low-nutrient conditions and treated with *S. plymuthica* MBSA-MJ1 had significantly higher concentrations of certain nutrients in the shoot tissue; however, differences varied depending on plant species (Figures 3, 4). Petunia, impatiens, and pansy plants had an increase of 32, 74, and 82% in tissue nitrogen concentration when treated with MBSA-MJ1, respectively. No other nutrients were significantly greater in petunia plants treated with MBSA-MJ1 when compared to the control. The concentrations of potassium, calcium, and sulfur were significantly greater in bacteria-treated impatiens and pansy plants. Only impatiens showed a significant increase in magnesium, boron, copper, molybdenum, and zinc when treated with MBSA-MJ1. Phosphorus was the only element where an increase was only observed in pansy. No significant differences in iron concentration were observed in any of the three plant species, and manganese concentration was found to be significantly greater in the control plants of petunia and pansy compared to those treated with MBSA-MJ1.

### *In vitro* Characterization

*S. plymuthica* MBSA-MJ1 was able to grow on basal media containing either cellobiose, fructose, galactose, glucose, glycerol, mannitol, sucrose, or ribose as the sole carbon source, indicating its ability to utilize each as an energy source.

*In vitro* characterization of MBSA-MJ1’s ability to increase the availability of nutrients showed that the strain was able to solubilize phosphate and potassium. Media containing insoluble forms of phosphorus and potassium had a significant decrease in pH when inoculated with MBSA-MJ1 compared to the uninoculated control (Table 1). The pH of the media decreased by 29 and 32% for the phosphate and potassium solubilization assays when inoculated with MBSA-MJ1, respectively. In addition, defined media with insoluble forms of phosphorus or potassium had a significant increase in available orthophosphate and potassium after inoculation with MBSA-MJ1 (Table 1). MBSA-MJ1 was also able to chelate copper and iron *in vitro* as indicated by sample color change when evaluated with the CAS assay. Absorbance values of the bacteria and control samples converted to chelation percentages show that MBSA-MJ1 was able to chelate over 62 and 63% of the iron and copper in the media, respectively (Table 1).

**Table 1:** *In vitro* characterization of nutrient solubilization and chelation abilities of *S. plymuthica* MBSA-MJ1.

	Phosphate solubilization^z^		Potassium solubilization^z^	Iron chelation^y^		Copper chelation^y^
	pH	Solubilized PO_4_ (mg/L)	pH	Solubilized K (mg/L)	Chelation (%)	Chelation (%)
MBSA-MJ1	4.32 b	150.40 a	4.66 b	4.07 a	62.17	63.36
Control	6.08 a	11.33 b	6.89 a	1.74 b	–	–

z The pH and concentration (mg/L) of solubilized PO_4_ and K were measured in defined media containing insoluble forms of phosphorus and potassium, respectively, and inoculated with MBSA-MJ1 or the uninoculated control.

y Iron and copper chelation percentages were calculated from the CAS assay using absorbance values of samples inoculated with MBSA-MJ1 and the negative control.

Values in each column with different letters indicate statistically significant differences.

### Genomic Analyses to Identify Genes Putatively Involved in Growth Promotion

#### Nitrogen Availability and Transport

Annotation files for *S. plymuthica* MBSA-MJ1 were used to identify genes within the genome that are putatively involved in nutrient metabolism and increasing availability to plants (Supplementary Table S1). Genes encoding for the nitrogen starvation transcription regulation system that are also involved in ammonium transport (*glnD*, *glnK*, and *amtB*) were identified in the genome of MBSA-MJ1(Supplementary Table S1). The genome of MBSA-MJ1 encodes for genes involved in nitrate transport (*nrtA*), nitrite uptake and reduction to ammonium (*nirC*), nitrate reduction (*nasA*), nitrite reduction (*nirD*) and its cofactor (*cysG*), and the operons responsible for nitrate and nitrite reductase (*narLXKGHJI*) and fumarate reductase (*frdABCD*; Supplementary Table S1). Genes involved in nitrogen assimilation (*gdhA*, *glnA*, *glnB*, *glnD*, *glnL*, *gltB*, and *gltD*) and periplasmic nitrate reductase (*napA*) were also identified (Supplementary Table S1).

#### Phosphate Availability and Transport

Bacteria can convert phosphorus to inorganic bioavailable forms *via* nonspecific phosphatases, phytases, and phosphonatases (Liu et al., 2018). The genome of MBSA-MJ1 encodes for the *appA* enzyme, which has both acid phosphatase and phytase activity, a polyphosphate kinase (*ppk*), exopolyphosphatases (*ppx* and *gppA*), a pyrophosphatase (*ppa*), alkaline phosphatase (*phoA*), and a nonspecific acid phosphatase (*phoC*; Supplementary Table S1). Genes encoding for both low-affinity (*pitA*) and high-affinity phosphate transporters (*phnCDE_1_E_2_* and *pstSCAB*) were identified within the genome (Supplementary Tables S1 and S3). We identified components of the bacterial P signaling pathway including the phosphate starvation two-component system (*phoBR*) and the negative regulator of phosphate transport (*phoU*; Supplementary Table S1). Genes involved in the catabolism of phosphonates and phosphites (*phnGHIJKLMOP*) and the negative regulator of PhnCDE (*phnF*) were also identified (Supplementary Table S1).

#### Zinc Availability and Transport

The MBSA-MJ1 genome encodes for the high-affinity ABC zinc transporter (*znuABC*) and its regulator (*zur*), which are responsible for zinc transport under low-zinc conditions (Supplementary Table S1). Genes encoding for both constitutive (*zitB*) and regulated (*zntABR*) zinc export were also identified (Supplementary Tables S1 and S3).

#### Organic Acid Synthesis

Mineral nutrient solubilization is most often attributed to the production of organic acids (Rodríguez and Fraga, 1999; Rodríguez et al., 2006). Genes involved in the metabolism of gluconic, ketogluconic, acetic, glyoxylic, lactic, and glycolic acid were identified (Supplementary Table S1). In particular, the genome included multiple genes putatively involved in the synthesis of gluconic acid (*pqqB*, *pqqC*, *pqqD*, *gcd*, *gdh*, *gnl*, *kdgK*, *gnd*, *gntR*, and *ylil*; Supplementary Table S1).

#### Sulfur Availability and Transport

The genome of MBSA-MJ1 encodes for the master regulator and transcriptional activator under sulfur starvation (*cysB*) and the sulfate ABC-type transporter complex (*sbp* and *cysPWAT*; Supplementary Table S1). The operon responsible for alkanesulfonate transport under sulfur limiting conditions (*ssuACBDE*), which includes an ABC-like transport system, was also identified (Supplementary Table S1). Additionally, the genome encodes for multiple genes involved in sulfur metabolism (*cysND*, *cysC*, and *cysHIJ*; Supplementary Tables S1 and S3).

#### Iron Chelation and Transport

The antiSMASH analysis predicted two biosynthetic gene clusters that encode for the siderophores malleobactin and amonabactin (Supplementary Table S2). The genome of MBSA-MJ1 also encodes for other genes involved in iron transport, such as the two-component system to transport ferric iron (*basSR*), the operon responsible for ferric hydroxamate uptake (*fhuADCB*), the ferrous iron transporters (*feoABC* and *efeUOB*), and two copies of the ferrous ion efflux pump (*fieF*; Supplementary Table S1). Additionally, the KEGG pathway analysis identified ABC transporters for iron (II; *sitACDB*), iron (III; *afuABC*), iron-enterobactin complex (*fepBDGC*), and the iron (III) hydroxamate complex (*fhuDBC*; Supplementary Table S3).

#### Amino Acid and Carbon Metabolism

KEGG pathway analysis identified genes responsible for the synthesis of several amino acids within the genome of MBSA-MJ1, including isoleucine, valine, leucine, lysine, arginine, proline, phenylalanine, tryptophan, and tyrosine. Further, amino acid uptake systems were identified for tryptophan, tyrosine, phenylalanine (*aroP*), isoleucine (*brnQ*), asparagine (*gltP*), glutamate (*gltP* and *gltS*), and serine and threonine (*sstT*; Supplementary Table S1). ABC transporters were also identified for lysine/arginine/ornithine (*argT* and *hisMQP*), histidine (*hisJMQP*), glutamine (*glnHPQ*), arginine (*artJMIQP*), glutamate/aspartate (*gltIKJL*), cystine (*tcyABC*), methionine (*metQIN*), proline (*proVWX*), and branched-chain amino acids (*livKHMGF*; Supplementary Table S3). In addition to amino acids, genes were identified for both the transport and metabolism of a variety of sugars (Table 2).

**Table 2:** Genes identified in the genome of *S. plymuthica* MBSA-MJ1 involved in sugar transport and metabolism.

Sugar	Transport	Metabolism
Arabinose	*araE*	*araBAD*
Arabinogalactan	*cycB*	*ganB*
Galactose	*galP*	*galK, galT*
Glycerol	*glpF*	*glpK, glpD*
Lactose	*lacY*	*lacZ*
Maltose/Maltodextrin	*malGFEK*	*malP, malQ, malS, malZ*
Melibiose, raffinose, and sucrose	*scrY*	*rafA, rafR, scrB, scrK*
Ribose	*rbsACB, rbsD*	*rbsK, rbsD, rbsR*
Xylose	*xylFGH*	*xylAB*
Cellulose and cellobiose	–	*bcsZ, bglB, yliI*
Citric acid	*citT*	*citABCDEFXG*
Glucose	*galP, glk*	*pgm, yihX, glk, pgi*
Glycosides	–	*bglB, bglA, ascG*

#### Heavy Metal Resistance

Multiple copper resistance proteins were identified (*copA*, *cueO*, *cueR*, and *pcoC*; Supplementary Table S1). Additionally, the genome of MBSA-MJ1 encodes for two lead/cadmium/zinc/mercury resistance proteins (*zntA* and *zntR*), two copies of the chromate resistance protein (*chrR*), two nickel transporters (*hypA* and *hypB*), and the two-component system involved in response to heavy metals (*basRS*; Supplementary Table S1).

## Discussion

Although the benefits of fertilizer application are easily recognizable in agricultural production systems, excessive application of these chemicals has negative effects on the environment. Negative environmental impacts of fertilization can often be attributed to low bioavailability and uptake by plants, leaving excess nutrients more prone to leaching (Yang et al., 2009). Therefore, it is important for crop producers to have sustainable options to ensure plants have an adequate supply of nutrients for proper growth and development, without relying on increasing applications of chemical fertilizers. We used a rate of 25mgL^−1^ N from 15N–2.2P–12.5K–2.9Ca–1.2Mg fertilizer in our greenhouse experiments because it is less than 20% of the average recommended fertilizer rate for ornamental crop species (150–200mgL^−1^ N; Beytes and Hamrick, 2003). The application of biostimulant products containing plant growth promoting bacteria (PGPR) with the ability to increase the bioavailability of nutrients provides a sustainable approach for growers to ensure proper nutrient supply while reducing chemical inputs (Saravanan et al., 2007; Park et al., 2009; Kraiser et al., 2011; Gupta et al., 2015; Ruzzi and Aroca, 2015; Drobek et al., 2019; Kramer et al., 2020). In this work, we determined that *S. plymuthica* MBSA-MJ1 can increase the growth and quality of containerized horticulture crops produced with low fertilizer inputs. Our work has utilized the biological and genomic characteristics of this beneficial bacteria to identify different putative mechanisms that MBSA-MJ1 may utilize to increase the availability of nutrients to plants and promote growth under otherwise limiting conditions.

The results obtained from the low-nutrient greenhouse trial provide evidence that MBSA-MJ1 can significantly improve the growth and quality of different plant species grown under low-nutrient conditions (Figures 1, 2). Multiple factors influence host specificity of both endophytic and rhizospheric bacteria, including attraction to plant root exudates, root colonization, and functioning of plant growth promoting mechanisms, contributing to varied host specificity between strains (Drogue et al., 2012; Tabassum et al., 2017; Afzal et al., 2019). Broad host specificity is particularly important for application in greenhouse production, which is characterized by the production of many different plant species. Although the application of MBSA-MJ1 only increased the flower number of impatiens compared to the uninoculated control, application did significantly increase the shoot biomass of all three plant species. Similarly, previous work with the same plant species showed application of *Pseudomonas poae* 29G9 or *Pseudomonas fluorescens* 90F12-2 increased the flower number of impatiens, while also increasing the total biomass of petunia, impatiens, and pansy plants grown under similar low-nutrient conditions (Nordstedt et al., 2020).

The growth differences observed between plants treated with MBSA-MJ1 and the uninoculated control are likely a result of MBSA-MJ1’s ability to increase plant nutrient levels. This is particularly apparent with the difference in the tissue nitrogen concentration, where all three plant species had a very large increase in foliar nitrogen when treated with MBSA-MJ1 (Figure 3A), with up to 82% more nitrogen in pansy. This is similar to other reports that show *Serratia* spp. increase tissue nitrogen and leaf chlorophyll content compared to uninoculated control plants (Martínez et al., 2018). Due to high concentrations of carbon but low concentrations of other nutrients in soilless media, microorganisms have the potential to deplete available nitrogen sources leading to plant nutrient deficiency (Jackson et al., 2009). Nitrogen deficiency in plants often leads to a decrease in leaf chlorophyll content, and therefore a yellowing of the leaves (Boussadia et al., 2010). In our study, leaf yellowing was observed in the uninoculated control plants, but not in the three plant species treated with MBSA-MJ1, even though all plants were grown under similar low-nutrient conditions (Figure 1). Although bacterial nitrogen fixation can be very complex, our genomic analyses identified a variety of genes involved in ammonium, nitrate, and nitrite transport and reduction that likely play a role in MBSA-MJ1’s ability to increase bioavailable nitrogen to the plant when grown under low-nutrient conditions, contributing to the growth promotion observed in the greenhouse trial.

Following nitrogen, phosphorus is the second most important element in plant nutrition. Although phosphorus is often present in abundant amounts, the total phosphorus that is available to plants is typically at very low concentrations due to its poor solubility (Gupta et al., 2015; Wang et al., 2018; Borgi et al., 2020). Phosphorus is usually applied in excess in greenhouse production and is more easily leached from the soilless substrates, leading to increasing environmental pollution (Kim and Li, 2016). Bacterial phosphate solubilization has been widely accepted as a mechanism for PGPR to increase phosphorus bioavailability to plants (Rodríguez et al., 2006; Park et al., 2009; Chen et al., 2014; Goswami et al., 2016), and *Serratia* spp. have been reported to be phosphate solubilizers (Ben Farhat et al., 2009). Our *in vitro* characterization of MBSA-MJ1 showed that the strain is an efficient phosphate solubilizer, as indicated by its ability to convert insoluble phosphorus to bioavailable orthophosphate in defined media (Table 1). The tissue nutrient analyses confirmed that this increased availability resulted in increased total phosphorus in the shoots of pansy plants (Figure 3B). Similarly, *S. plymuthica* BMA1 is an efficient phosphate solubilizer that increased plant growth of *Vicia faba* (Borgi et al., 2020). The pH reduction observed in the *in vitro* phosphate solubilization assay (Table 1), coupled with the identification of multiple genes involved in gluconic acid synthesis (Silva et al., 2021), indicates that MBSA-MJ1 could be producing organic acids as a mechanism to solubilize phosphate (Rodríguez et al., 2006). This mechanism for phosphate solubilization has been previously reported in *Serratia marcescens* strains (Chen et al., 2006; Selvakumar et al., 2008; Ben Farhat et al., 2009). Impatiens and pansy plants treated with MBSA-MJ1 also had significantly greater tissue potassium concentration than the uninoculated control (Figure 3C). Application of *Serratia marcescens* strains NBRI1213 and MTCC 8708 similarly show increases of phosphorus and potassium tissue nutrient content in maize and wheat plants (Selvakumar et al., 2008; Lavania and Nautiyal, 2013). Our *in vitro* characterization showed a similar decrease in media pH and increase in soluble potassium in defined media when inoculated with MBSA-MJ1 (Table 1); therefore, it is probable that MBSA-MJ1 is able to utilize organic acid production as a mechanism to solubilize and increase the bioavailability of potassium similar to phosphate (Sharma et al., 2016).

Zinc is essential to many plant physiological processes including chlorophyll synthesis, and zinc deficiency can negatively impact plant growth and development (Hussain et al., 2015). In our work, we showed that impatiens grown under low-nutrient conditions had significantly higher levels of zinc in shoot tissues when treated with MBSA-MJ1 as compared to the uninoculated control (Figure 4F). Bacterial zinc solubilization is one mechanism that can increase the bioavailability of this valuable nutrient to plants, and research has shown that zinc solubilizing bacteria can increase plant growth (Ramesh et al., 2014; Kamran et al., 2017). Similar to phosphate and potassium, zinc becomes more soluble and available to plants at a lower pH (Hussain et al., 2015). Research has shown that phosphate solubilizing bacteria can also increase plant zinc uptake (Salimpour et al., 2012). Considering MBSA-MJ1’s ability to solubilize phosphate and potassium through a reduction in pH, the production of organic acids by MBSA-MJ1 is a potential mechanism allowing this strain to solubilize phosphorus, potassium, and zinc, making them more bioavailable and increasing tissue nutrient levels.

Iron is usually present in abundant amounts in soil, but its availability to plants is often dependent on the production of iron chelators, such as bacterial-produced siderophores (Powell et al., 1980). Microbial-produced siderophores have been shown to increase iron acquisition and reduce iron deficiency symptoms in multiple crops grown under iron-deficient conditions (Sharma et al., 2003; Jin et al., 2006; Zhou et al., 2018; Lurthy et al., 2020). Our *in vitro* characterization showed that MBSA-MJ1 acts as an efficient iron chelator (Table 1). Additionally, genomic analyses identified biosynthetic gene clusters for two siderophores and multiple genes involved in bacterial iron transport and metabolism. However, our tissue nutrient analysis did not show any significant differences between bacterial-treated plants and the control (Figure 4C). Lurthy et al. (2020) suggests that the impact of siderophores on plant iron acquisition is highly dependent on the type of siderophore and the plant host. Therefore, it is probable that MBSA-MJ1 could assist in iron acquisition when colonizing plant hosts not evaluated in this study.

Copper is another essential metal element; however, it can be toxic to cells above certain levels (Vita et al., 2016). Our tissue nutrient analysis showed that impatiens plants treated with MBSA-MJ1 had significantly higher copper levels than the untreated control. However, the plants did not exhibit any signs of copper toxicity, such as stunted growth or the inhibition of photosynthesis, as these plants were larger and greener than untreated plants (Figures 1, 2). The optimal tissue copper concentration in impatiens is 10–15μgg^−1^ (Dole and Wilkins, 1999). The control plants had only 10μgg^−1^, whereas plants treated with MBSA-MJ1 had 13.7μgg^−1^ (Figure 4B). This indicates that MBSA-MJ1 can increase levels of this essential plant nutrient without reaching toxic levels. Our *in vitro* characterization of MBSA-MJ1 showed that like iron, the strain could efficiently chelate copper (Table 1). This likely provides a mechanism for MBSA-MJ1 to provide safe levels of copper to its plant host and augment growth under low-nutrient conditions. Notably, our genome analyses also identified four copper resistance proteins that may be involved in protecting the bacteria from potentially toxic exposure to copper (Supplementary Table S1; Lee et al., 2002).

Recent work has shown that application of sulfur oxidizing bacteria increases soil sulfate concentration and increases plant growth and sulfur uptake in maize and garlic (Youssif et al., 2015; Pourbabaee et al., 2020). In our study, we observed that impatiens and pansy plants grown under low-nutrient conditions and treated with MBSA-MJ1 had significantly greater levels of sulfur when compared to the uninoculated control (Figure 3F). Additionally, our genomic analyses identified both the master transcriptional regulator under sulfur starvation (*cysB*) and the sulfate transporter complex (*sbp*-*cysPWAT*) encoded in the genome of MBSA-MJ1 (Supplementary Table S1). These genes could potentially play a role in MBSA-MJ1’s ability to sense limited sulfur in the rhizosphere and supply bioavailable sulfur to the plant host, corroborating the increase in plant tissue concentration that was observed.

In addition to the nutrients already discussed in detail, it should be noted that treatment with MBSA-MJ1 significantly increased the tissue concentration of other essential nutrients, such as boron, calcium, magnesium, and molybdenum (Figures 3, 4). Impatiens and pansy treated with MBSA-MJ1 also had significantly greater tissue sodium concentration (data not shown). Interestingly, the untreated plants had higher manganese content than bacterial-treated plants (Figure 4E). When plants are grown under phosphorus-deficient conditions, as were induced by our low fertilizer treatment, many plant species increase root exudation of carboxylates, which increase the availability and uptake of manganese (Lambers et al., 2014). Bacteria that increase the availability of phosphorus in the rhizosphere may prevent the release of carboxylates from the roots and subsequently these plants will have reduced Mn uptake and Mn tissue concentration. Although the emphasis on understanding bacterial nutrition-related plant growth promoting mechanisms have focused on nutrients, such as nitrogen and phosphorus, our work has shown the bacterial application can increase the tissue levels of these other important macro and micronutrients. Therefore, these results provide justification for future work to begin understanding bacterial mechanisms involved in increasing the availability or uptake of nutrients to plants grown under low-nutrient conditions.

Amino acids and carbon secreted by plant roots serve as an important method to recruit and sustain beneficial rhizospheric bacteria. Therefore, bacteria with robust amino acid and carbon source metabolism are more likely to persist in the rhizosphere and be able to positively influence the growth of their host (Lugtenberg and Kamilova, 2009). Genomic analyses conducted in this work identified a series of genes related to amino acid and carbon metabolism and transport. In addition to the genomic analyses, our *in vitro* characterization assays provided evidence that MBSA-MJ1 could utilize a variety of carbon sources for growth. Not only is this robust carbon metabolism useful for persistence in the rhizosphere, but it also has the potential to increase MBSA-MJ1’s host range, a valuable trait for potential commercial biostimulant products.

## Conclusion

*Serratia plymuthica* MBSA-MJ1 significantly increased the shoot biomass of all three plant species, the flower number of impatiens plants, and the tissue concentrations of certain nutrients in different plant species grown under low-nutrient conditions. In addition, the comprehensive genomic analyses shed light on different genes encoded within MBSA-MJ1’s genome that are putatively involved in mechanisms conferring plant growth promotion under low-nutrient conditions. Plant growth promotion by rhizospheric bacteria is likely the result of multiple coordinated mechanisms, and this work begins to highlight how interconnected mechanisms can increase overall plant health and quality. Increasing plant health under low-nutrient conditions without increasing chemical inputs provides an exciting option for crop producers to improve environmental sustainability. Formulation of biostimulant products containing well-characterized PGPR is a viable solution to accomplish this goal. Using MBSA-MJ1 as a model strain, future work should be invested in characterizing how PGPR increase the bioavailability or uptake of different essential nutrients by plants. Our work has defined the biology and genomic characteristics of this agriculturally important PGPR, contributing to a comprehensive understanding of this strain’s ability to increase plant growth under low-nutrient stress and the mechanisms that might be allowing this interaction to take place.

## Data Availability Statement

The datasets presented in this study can be found in online repositories. The names of the repository/repositories and accession number(s) can be found at: https://www.ncbi.nlm.nih.gov/genbank/, PRJNA669647.

## Author Contributions

NN and MJ conceived the project and experimental design. NN performed the greenhouse production trial, *in vitro* assays, genomic analyses, and led the writing of the manuscript. MJ acquired funding and resources, served as project administrator, and edited the manuscript. All authors contributed to the article and approved the submitted version.

## Funding

Salaries and research support were provided in part by the State and Federal funds appropriated to the OARDC, the Ohio State University. Journal Article Number HCS 21–01. This work was financially supported, in part, by the American Floral Endowment, the Floriculture and Nursery Research Initiative, USDA-ARS cooperative agreement #58–5082–0-006 as part of the Application Technology Research Unit project #5082–21000-001-00D, and the OSU D.C. Kiplinger Floriculture Endowment. Support was also provided to NN by the Ohio State University Distinguished Fellowship, the OARDC Director’s Graduate Associateship, and the Altman Family Scholarship.

## Conflict of Interest

The authors declare that the research was conducted in the absence of any commercial or financial relationships that could be construed as a potential conflict of interest.

## Publisher’s Note

All claims expressed in this article are solely those of the authors and do not necessarily represent those of their affiliated organizations, or those of the publisher, the editors and the reviewers. Any product that may be evaluated in this article, or claim that may be made by its manufacturer, is not guaranteed or endorsed by the publisher.
